# Signal processing and stimulation potential within the ascending auditory pathway: a review

**DOI:** 10.3389/fnins.2023.1277627

**Published:** 2023-11-03

**Authors:** Alexandra E. Quimby, Kimberly Wei, Dayo Adewole, Steven Eliades, D. Kacy Cullen, Jason A. Brant

**Affiliations:** ^1^Department of Otolaryngology and Communication Sciences, SUNY Upstate Medical University, Syracuse, NY, United States; ^2^Perelman School of Medicine, University of Pennsylvania, Philadelphia, PA, United States; ^3^Corporal Michael J. Crescenz Veterans Affairs Medical Center, Philadelphia, PA, United States; ^4^Department of Head and Neck Surgery and Communication Sciences, Duke University, Durham, NC, United States; ^5^Department of Otorhinolaryngology – Head and Neck Surgery, University of Pennsylvania, Philadelphia, PA, United States

**Keywords:** central auditory processing, auditory pathway, optogenetics, auditory signal processing, cochlear implant – neuroprosthesis

## Abstract

The human auditory system encodes sound with a high degree of temporal and spectral resolution. When hearing fails, existing neuroprosthetics such as cochlear implants may partially restore hearing through stimulation of auditory neurons at the level of the cochlea, though not without limitations inherent to electrical stimulation. Novel approaches to hearing restoration, such as optogenetics, offer the potential of improved performance. We review signal processing in the ascending auditory pathway and the current state of conventional and emerging neural stimulation strategies at various levels of the auditory system.

## 1. Introduction

The auditory system is a multi-level sensorineural pathway that transforms acoustic waves into neural signals which are experienced as sound. The pathway, in brief, is as follows: sound pressure waves enter the outer ear and reach the tympanic membrane; the resulting movement of the membrane induces vibration of the middle ear ossicles. The middle ear system concentrates the incoming sound waves as mechanical energy to match the impedance encountered as it is transferred from the air into the incompressible cochlear fluids of the inner ear. Energy transfer within the cochlea induces hair cell depolarization, resulting in auditory nerve firing, with the resultant action potential traveling from the cochlear nerve to the brainstem nuclei, from whence it ascends along the central auditory pathway to eventually reach auditory cortex where the sound is perceived. The central auditory pathway consists of multiple levels of nuclei and tracts where the signal is processed as well as influenced by contralateral pathways, descending inputs, and non-auditory signals (Flint et al., 2020).

Hearing loss is one of the most pervasive sensory disorders with approximately 430 million people affected worldwide. Disabling hearing loss often leads to social isolation, and has been associated with depression, incident dementia, and reduced quality of life (Uhlmann et al., 1989; Strawbridge et al., 2000; Kramer et al., 2002; Viljanen et al., 2009; Gopinath et al., 2012; All Ears International, Vaughan G, 2023). The etiopathology in most cases of sensorineural hearing loss (SNHL) is hair cell loss or dysfunction at the level of the cochlea, which may be secondary to a variety of mechanisms. Principal among these are age- and noise-related hair cell loss, mediated at a cellular level largely by reactive oxygen species (Kamogashira et al., 2015). Rehabilitation is largely accomplished through amplification to overcome hair cell loss. However, in cases of severe to profound hearing loss, especially when speech perception is significantly reduced, the role for traditional hearing aid amplification is limited.

The cochlear implant (CI) has consistently been demonstrated to significantly improve hearing, communication, and quality of life in individuals with severe to profound SNHL. Cochlear implants bypass cochlear dysfunction by delivering electrical current from within the cochlear scalae that results in activation of spiral ganglion neurons (SGNs) which is then transmitted up the ascending auditory pathway. Most adults implanted with a CI achieve good hearing performance outcomes, as measured by open-set speech perception (Holden et al., 2013). However, the performance of cochlear implants in complex noisy environments and in response to music is negatively affected by the limited number of independent channels achievable with current technology, which is inherent to the delivery of current within a liquid medium, as well as challenges in encoding sound intensity (McDermott and McKay, 1997; Zeng et al., 2002; Crew et al., 2012; Dieter et al., 2020). Additionally, cochlear implants are not a feasible option in individuals with absent or non-functioning auditory nerves — whether this be congenital, traumatic, or secondary to neoplasm — and may not be effective for dysfunction of higher levels of the auditory pathway. In such cases, the only currently available alternative for hearing restoration is the auditory brainstem implant (ABI), which delivers current at the level of the cochlear nucleus; its use, however, is predicated on the integrity of more proximal components of the auditory pathway. The performance of ABIs has been limited and auditory perception is generally inferior to that achieved by cochlear implants (Colletti et al., 2009; Deep and Roland, 2020).

Given the limitations of currently available implantable devices for hearing rehabilitation, the development of novel approaches for the treatment of hearing loss has been an area of active investigation. The concept of direct neural stimulation by infrared light has evolved into the study of optogenetics in the auditory system (Dieter et al., 2020). Here, we review signal processing in the central auditory pathway, as well as stimulation potential and limitations. Novel hearing restoration strategies are reviewed, with a focus on optogenetic stimulation of the auditory pathway. Cochlear gene therapy is another potential avenue of treatment, but is beyond the scope of this review.

## 2. Signal processing

### 2.1. Cochlea and auditory nerve

After being perpetuated as mechanical energy through the outer and middle ear, sound reaches the cochlea, where it is transformed into an electrical neural signal at the level of the inner hair cell. Stapes movements in the oval window produce fluid waves in the cochlear scalae that propagate down the basilar membrane. Shearing forces on hair cell stereocilia induced by pressure differentials across the cochlear partition lead to the opening or closing of mechanically-gated ion channels and resultant cellular depolarization or hyperpolarization. This process of hair cell mechanoelectrical transduction involves the generation of receptor potentials as a result of ion flow, with the response consisting of an alternating current (AC) component encoding sound frequency and amplitude, as well as a direct current “summation potential” (SP) generated by potassium flow at the level of the hair cell. The SP may have a positive or negative polarity which is measurable and may vary in healthy and disease states (Fettiplace, 2017; Hazkizimana, 2023). Indeed, computational methods have been developed to isolate the SP component from electrocochleography (ECoG) recordings in order to differentiate hair cell and auditory nerve pathology (Vasilkov et al., 2023). The aforementioned specializations allow the cochlea to encode sounds with a high degree of temporal precision: current changes can be detected within microseconds of the onset of a sound stimulus (Hudspeth, 1979). Sound frequency is represented by the position of hair cells and SGN fibers along the length of the cochlea, which in turn is dictated by the physical properties of the basilar membrane – which vary apico-basally – and are further fine-tuned by active mechanical amplification from the outer hair cells (Johnson et al., 2019). High frequencies (up to 20 kHz in humans) are encoded by hair cells at the cochlear base, where the basilar membrane is narrow, thick, and stiff; low frequencies (≥ 20 Hz) are represented at the apex, where the basilar membrane is widest and least stiff (Johnstone et al., 1986). Outer hair cells contribute to the precise “tuning” of the basilar membrane, amplifying the degree of displacement in a given region in response to sounds of that region’s resonant or characteristic frequency (*CF*). The result of this cochlear tonotopic organization is our ability to resolve tones that differ in frequency by only 0.2% (Hudspeth, 2014). Tonotopy is preserved within SGNs of the auditory nerve by their organized hair cell connectivity and other unique tonotopic specializations – including morphology and firing features – allowing preservation of sound frequency information to be transmitted to the cochlear nucleus (Young, 2007). SGN neurons respond best (ie, are most sensitive in their responses) to tones at their *CF* – the so-called “place principle.” Notably, however, such place-coding appears not to be true for SGNs innervating the apical 20% of the cochlea. Rather, low-frequency pitch perception relies on the timing of action potentials in the auditory nerve, and to this end outer hair cell activity is critical in distributing excitation to large numbers of sensory cells (Burwood et al., 2022). In addition to place coding of the spectral contents of sound, auditory nerve fibers also utilize firing rate to encode information about sound intensity and relative timing, and phase synchronization (phase locking) to encode temporal information (Sachs, 1984). Phase locking – which refers to the ability of SGNs to synchronize or “lock” their firing to typical and consistent points (phase) within the neuronal response to a sinusoidal sound pressure waveform – is key in encoding accurate representations of the spectral shape of sounds (Young and Sachs, 1979). However, phase locking has been shown to occur only at simulation frequencies below approximately 3 kHz (Palmer and Russell, 1986; Peterson and Heil, 2019). Phase locking also occurs at the level of cortical auditory neurons (Peelle et al., 2013).

### 2.2. Cochlear nucleus

At the level of the pontomedullary junction along the floor of the lateral recess of the fourth ventricle, SGNs of the auditory nerve branch to innervate two subdivisions of the ipsilateral cochlear nucleus: the dorsal cochlear nucleus (DCN) and ventral cochlear nucleus (VCN). The tonotopic organization established in the cochlea is again reflected in the cochlear nucleus: neurons whose CFs range from high to low are organized from dorsal to ventral in the VCN, and from dorsomedial to ventrolateral in the DCN (Osen, 1970). Each of the principal cell types of the cochlear nucleus, whose compositions vary across the DCN and VCN, receive input from SGNs across the entire frequency range (Mugnaini et al., 1980). These cell types’ differing electrical and physical properties, as well as their unique patterns of innervation by auditory nerve fibers, contribute to each encoding disparate components of the auditory signal (Oertel et al., 2011). Neural outputs from the cochlear nuclei project to other brainstem regions via the dorsal, intermediate, and ventral acoustic striae (trapezoid body). Of these, the ventral acoustic striae (VAS) is the major component of the ascending auditory system (Masterton and Granger, 1988; Sutherland et al., 1998). Upstream targets include the medial and lateral superior olivary nuclei (MSO, LSO), the medial and lateral nuclei of the trapezoid body (MNTB, LNTB), and the inferior colliculus (IC) (Mugnaini et al., 1980). The cochlear nucleus is one of the first sites to receive direct modulating signals from descending auditory pathways, including from the somatosensory, vestibular, and auditory cortices (Osen, 1970; Kandler et al., 2009). However, both the cochlea and middle ear muscles have also been shown to receive descending input from the auditory brainstem (Mukerji et al., 2010; Romero and Trussell, 2022).

### 2.3. Superior olivary complex

The superior olivary complex (SOC) – whose subdivisions include the LSO, MSO, and MNTB – extends from the rostal medulla to the caudal pons and is the first site of convergence of auditory input from both ears. Tonotopic arrangement of auditory inputs is again maintained at this level of the pathway, though neuronal response patterns to sound stimuli are more complex and varied (Guinan et al., 2009; Tabor et al., 2012). Comparisons of ipsilateral and contralateral inputs contribute to sound localization ability, with neurons in the MSO assessing interaural time differences and those in the LSO receiving converging input concerning interaural latency differences (Grothe, 2000; Tollin, 2003). Descending projections originating at the level of the SOC reach the cochlear nucleus posteroventrally to provide cochlear protection from acoustic overexposure and cochlear fine regulation which contributes to sound processing in noisy environments (Fekete et al., 1984; LePage, 1989).

### 2.4. Inferior colliculus

The inferior colliculus (IC) is the main center of convergence in the auditory midbrain. It receives and integrates diverse preprocessed inputs from all major auditory brainstem nuclei, as well as integrating auditory and non-auditory inputs. The IC is divided into three subdivisions: a central nucleus (ICC), external cortex, and dorsal cortex (Yang et al., 2020). The external and dorsal cortices receive both auditory and non-auditory inputs from descending projections (somatosensory and auditory cortices) as well as local inputs from the superior colliculus, while the ICC is the dominant region receiving direct ascending inputs from the auditory brainstem as well as the contralateral IC. In the ICC, tonotopy is again preserved, with neurons (and frequencies) distributed in a laminar organization (Merzenich and Reid, 1974; Stiebler and Ehret, 1985; Malmierca et al., 1995; Schreiner and Langner, 1997; Ress and Chandrasekaran, 2013). In addition to its critical role in integrating cues and information necessary for sound localization, IC neurons also exhibit more complex spectral and spectrotemporal integration important for the encoding of natural sounds (Lyzwa and Wörgötter, 2016). For example, connectivity of ICC neural circuits often cross multiple frequency laminae and, as a result, many receptive fields include broad frequency inputs as well as inhibitory sidebands that shape their sensory coding (Davis, 2005; Chen et al., 2018). Ascending fibers from the IC synapse at the medial geniculate body (MGB) of the thalamus.

### 2.5. Medial geniculate body and auditory cortex

The MGB receives most of its auditory input from the ICC, which it then distributes to the auditory cortex located within the superior temporal gyrus, as well as other non-auditory cortical regions. Ventral, dorsal, and medial divisions of the MGB each contain subnuclei receiving ascending and descending innervation from the IC and the auditory cortex (Winer, 1984). MGB inputs are amplified and further processed by local microcircuits, resulting in a refinement of neuronal frequency tuning (Liu and Kanold, 2021). The primary auditory cortex in turn receives point-to-point input from the ventral division of the MGB in a tonotopic manner, with responses further refined through processes of lateral connectivity and inhibition (Kato et al., 2017). As a result, neurons in the auditory cortex often have more complex receptive fields, including multi-peak frequency and harmonic tuning (Wang, 2013). Beyond spectral processing, auditory cortex neurons also exhibit encoding of both loudness and timing information that is more complex than that seen within more peripheral auditory areas (Arnal et al., 2015). For example, integration of neuronal signals occurs on different timescales in different areas of the auditory cortex, and there is evidence for hemispheric variation in temporal processing (Arnal et al., 2015). Higher order auditory and association areas beyond primary cortex may be involved in important processes like encoding of sound pitch (De Angelis et al., 2018), audiovisual integration (Chaplin et al., 2018), and receptive speech/language processing in Wernincke’s area (Koelsch and Siebel, 2005). Outputs of the auditory cortex are vast and include both corticocortical and corticothalamic pathways to regions including the amygdala, hippocampus, visual cortex, prefrontal cortex, as well as descending modulatory pathways (Saldaña, 1995; Mellott et al., 2014; Plakke and Romanski, 2014).

## 3. Stimulation strategies and limitations

### 3.1. Electrical stimulation

When hearing loss is beyond remedy by traditional hearing aids – for example, is severe to profound in degree, or with disproportionately poor speech recognition ability – the only widely available option for hearing restoration is a CI. Cochlear implants use an external processor use an external processor to mimic the spectral decomposition process of the normal cochlea, decoding sound stimuli into multi-frequency bands, and then provide electrical current stimulation through implanted intracochlear electrodes. Signal processing is accomplished through encoding of the speech envelope via continuous interleaved sampling (CIS) of input sound and speech waveforms (Wilson et al., 1991; Nuttall et al., 2018). CIS offers advantages over compressed analog (CA) processing by delivering temporally-offset trains of pulses (whose amplitudes are derived from the envelopes of filtered waveforms of the input signal) to each electrode; this interleaving of pulses reduces the channel interactions seen in CA processing (Wilson et al., 1991). Such channel interactions – which result from the overlap of electric fields from adjacent electrodes to which current is simultaneously delivered – may result in distorted neural responses in a CA processing strategy. Transmitted current stimulates SGNs directly, though with far less selectivity than is accomplished with natural cochlear transduction mechanisms. The resulting neural activity ascends through the auditory pathway and is interpreted by the auditory cortex as sound. This system relies on the integrity of both the auditory nerve and the remainder of the central auditory pathway. While the majority of CI recipients achieve open-set speech perception, challenges persist in the cochlear implant’s ability to allow accurate comprehension of speech in noise as well as music perception and enjoyment. Inherent to the use of electrical stimulation from within a liquid medium, current spread in perilymphatic fluids and the resultant channel interactions is a well-recognized limitation to CI frequency selectivity, which particularly limits listeners’ ability to comprehend speech in noise or discern closely spaced frequencies (Aronoff et al., 2016). The result of these channel interactions is that while modern cochlear implants may have up to twenty-two functioning individual electrode channels, the maximum useable at a given time is generally regarded to be eight (Friesen et al., 2001). Signal processing strategies such as CIS have been able to overcome these limitations, though with inconsistent performance benefits (Wilson et al., 1991; Mani et al., 2004; Aronoff et al., 2016). Pitch perception in CI users may also be detrimentally affected by the devices’ temporal encoding limitations: while it is likely that low pitches are encoded using phase locking by the CI, beyond 300–400 pulses per second (pps) higher rates of electrical stimulation are not perceived by listeners as corresponding increases in pitch (Carlyon et al., 2010; Tyler et al., 2010). Frequency resolution is also hampered by the limitations of cochlear place coding (as previously discussed), finite electrode counts, and current spread/channel interactions (Young and Sachs, 1979; Sachs, 1984; Palmer and Russell, 1986; Peelle et al., 2013; Peterson and Heil, 2019; Burwood et al., 2022). These limitations in both temporal and spectral resolution likely contribute to the difficulties experienced by CI users in perceiving and enjoying music (McDermott and McKay, 1997). The concept of place-pitch mismatch is another important consideration in this regard: mapping of representative input frequency information to inappropriate electrode contacts (which is based on the presumptive Greenwood map) may further limit speech perception and music enjoyment by CI users (Greenwood, 1990; Landsberger et al., 2015). Current spread and stimulation rate limitations also affect implants’ ability to accurately encode stimulus intensity; the dynamic range achievable by electrical stimulation is far less than that of a normal acoustically hearing ear (20 dB versus >100 dB) (Zeng et al., 2002; Flint et al., 2020). As a result, discrimination between higher levels of sound intensity is not possible using a CI.

When hearing loss is secondary to a defect or impairment of the auditory nerve, an auditory brainstem implant (ABI) is a feasible alternative. The ABI similarly relies on electrical current to produce neural activation. Implanted at the surface of the cochlear nucleus as a flat paddle, the neuronal activation induced by sound that is processed and transmitted via the device is even more crude as a result of both larger electrode contacts (relative to the size of the target structure) and the more nuanced tonotopic and cellular organization at this level of the auditory pathway – frequency selectivity is not achieved. ABI performance may also suffer from more complex intrinsic temporal responses seen in cochlear nucleus neurons, an encoding that is not easily reproduced by the implant stimulation. The prototypical population implanted with the ABI is patients with neurofibromatosis type 2 (NF2), in whom placement is performed concomitant to or following vestibular schwannoma removal. Open-set sentence perception ability is uncommon, though a majority of recipients able to achieve environmental sound awareness through use of the device (Colletti et al., 2009; Deep and Roland, 2020). By virtue of the location of the cochlear nucleus, placement of the device is also much more challenging and error-prone than cochlear implantation: the requisite craniotomy is not without attendant risks.

Emerging research has explored electrical stimulation of higher regions of the ascending auditory pathway. Direct stimulation of SGNs via an auditory nerve implant (ANI) has been proposed, though achieving frequency selectivity with an implant at this level of the auditory pathway should prove challenging (Dyballa et al., 2023; Nogueira, 2023). Such an implant would not be feasible in patients with a compromised auditory nerve (eg, NF2), and surgical access would require an approach similar to the ABI. A larger body of research has evaluated stimulation at the level of the ICC via an auditory midbrain implant (AMI) (Lenarz et al., 2003, 2005, 2006; Lim and Anderson, 2003, 2006). The device is designed as a penetrating electrode array that is implanted via a lateral supracerebellar infratentorial approach through a lateral suboccipital craniotomy. The penetrating design is intended to stimulate neurons along the tonotopic gradient of the ICC, which is possible in theory due to the region’s laminar tonotopy (Malmierca et al., 1995). In reality, the concept of frequency selectivity with a single penetrating electrode is likely an oversimplification in the context of the region’s varied neuronal composition and cross-laminar neuronal networks, as well as cross-tonotopic current spread in a brain area even more densely arranged than within the cochlea. In animal models, the AMI has been able to achieve a degree of frequency-specific activation as recorded in the primary auditory cortex (Lim and Anderson, 2003; Lenarz et al., 2005; Lim and Anderson, 2006). Performance of the implant in humans remains speculative.

As is exemplified by observed ABI outcomes and the theoretical challenges of the ANI and AMI, stimulation of increasingly proximal levels of the auditory pathway in a manner that results in an accurate neuronal representation of the precise spectral components of sound is infeasible with current approaches. The baso-apical tonotopy of the cochlea, uniform firing patterns of SGNs, and surgical ease of access make this first level of the auditory pathway the most attractive target for implantable auditory devices. However, there exist inherent limitations to electrical stimulation at the level of the cochlea, rendering an alternative means of SGN stimulation an appealing target of research.

### 3.2. Optical and optogenetic stimulation

Optical stimulation of auditory neurons using infrared light – or infrared neural stimulation (INS) – poses the theoretical advantage of improved spatial (and thus frequency) resolution. At high intensity levels, infrared light is thought to directly stimulate neurons to cause depolarization. A photoelectric effect, whereby heating of cochlear fluids generates fluid pressure waves akin to acoustic stimuli, may also contribute to neuronal responses to light stimulation within the cochlea (Thompson et al., 2014). Infrared optical stimulation of SGNs has been demonstrated in animal models to achieve high degrees of auditory frequency selectivity, though the requisite high energy requirements make practical applications largely untenable (Izzo et al., 2007, 2008; Matic et al., 2011; Richter et al., 2011).

The field of optogenetics involves the study of opsin genes in research; more recently, the field has been co-opted for clinical use in the restoration of vision, olfaction, and auditory perception. Channelrhodopsins (ChR), light-gated channels found in algae, render neurons light-sensitive; their expression can be induced in mammals by transfection of opsin genes using viral vectors (Arenkiel et al., 2007; Tomita et al., 2009). A variety of such opsin genes have been transfected in animal models, primarily rodents, often using adeno-associated viral (AAV) vectors. Following viral transfection, the proportion and apico-basal distribution of SGNs with ChR expression has varied, ranging from only 20–30% of SGNs to the majority of SGNs throughout all cochlear turns (Hernandez et al., 2014; Duarte et al., 2018; Wrobel et al., 2018). Light pulses delivered via optical fibers implanted in the cochleae of experimental animals with ChR expression are able to induce optically evoked potentials (oABRs) – the optically-induced correlate of the ABR, measured via scalp electrodes. High amplitude oABRs reflecting synchronous SGN firing have been recorded in response to a variety of stimulation frequencies (Keppeler et al., 2018; Mager et al., 2018; Wrobel et al., 2018). However, the inherent temporal kinetics of ChR and similar opsins still limit the temporal resolution of these approaches compared to the fine timing information used by the native auditory system. These approaches also continue to suffer from issues with dynamic range encoding. For example, multi-channel recordings of neuronal clusters in gerbil ICCs in response to acoustic, electrical, and optogenetic stimulation of SGNs demonstrated similar dynamic ranges with optical compared to electrical stimulation (10.7 dB versus 10.7/12.2 dB for monopolar/bipolar electrical stimulation), which were reduced compared to acoustic stimuli in non-implanted animals (32.2 dB). The spectral spread of excitation in response to optogenetic stimulation in this study demonstrated near physiologic frequency selectivity, which was improved compared to that achieved using electrical stimulation (Dieter et al., 2019). Behavioral correlates of optically-induced SGN stimulation have been observed in implanted mice (Wrobel et al., 2018). Optogenetic stimulation of more proximal levels of the central auditory system (cochlear nucleus) has also been shown possible in experimental animals, though is subject to similar limitations as electrical stimulation at proximal levels – that is, an inability to reproduce cellular specificity within complex receptive fields. The extent to which optogenetic stimulation may confer a superior representation of the precise spectral, temporal, and intensity features of an acoustic signal in humans remains speculative.

In addition to the limitations in stimulation specificity, other limitations of an optogenetic approach to hearing restoration arise from the use of viral vectors. When mice are transfected with opsin genes post-natally, protein expression is typically reduced compared to when transfected prenatally (intrauterine) (Duarte et al., 2018; Keppeler et al., 2018; Mager et al., 2018; Wrobel et al., 2018). Off-target gene expression is not uncommonly observed (Keppeler et al., 2018; Mager et al., 2018). In addition, the mechanism of viral transfection in humans – whether feasible via intratympanic injection or mandating surgical access to the inner ear – requires consideration.

## 4. Discussion

The tonotopic organization of the auditory system makes hearing restoration via implantable devices feasible; the CI is widely regarded as the most successful neural prosthesis in existence. However, physiologic limitations which curtail performance make the search for an alternative means of hearing restoration alluring. Devices that act to stimulate neurons at the level of the cochlea are likely to be most successful due to the increasing complexity of neuronal organization encountered at more proximal levels of the auditory pathway. Emerging research in the field of optogenetics suggests improved spectral selectivity compared to electrical neural stimulation. However, the practical feasibility of these approaches for hearing restoration in humans is as of yet unknown.

## Author contributions

AQ: Investigation, Methodology, Supervision, Writing – original draft, Writing – review & editing. KW: Investigation, Writing – original draft. DA: Writing – review & editing. SE: Writing – review & editing. DKC: Writing – review & editing. JB: Conceptualization, Supervision, Writing – review & editing.
